# A Role for Gene Duplication and Natural Variation of Gene Expression in the Evolution of Metabolism

**DOI:** 10.1371/journal.pone.0001838

**Published:** 2008-03-19

**Authors:** Daniel J. Kliebenstein

**Affiliations:** Department of Plant Sciences, University of California Davis, Davis, California, United States of America; University College Dublin, Ireland

## Abstract

**Background:**

Most eukaryotic genomes have undergone whole genome duplications during their evolutionary history. Recent studies have shown that the function of these duplicated genes can diverge from the ancestral gene via neo- or sub-functionalization within single genotypes. An additional possibility is that gene duplicates may also undergo partitioning of function among different genotypes of a species leading to genetic differentiation. Finally, the ability of gene duplicates to diverge may be limited by their biological function.

**Methodology/Principal Findings:**

To test these hypotheses, I estimated the impact of gene duplication and metabolic function upon intraspecific gene expression variation of segmental and tandem duplicated genes within *Arabidopsis thaliana*. In all instances, the younger tandem duplicated genes showed higher intraspecific gene expression variation than the average Arabidopsis gene. Surprisingly, the older segmental duplicates also showed evidence of elevated intraspecific gene expression variation albeit typically lower than for the tandem duplicates. The specific biological function of the gene as defined by metabolic pathway also modulated the level of intraspecific gene expression variation. The major energy metabolism and biosynthetic pathways showed decreased variation, suggesting that they are constrained in their ability to accumulate gene expression variation. In contrast, a major herbivory defense pathway showed significantly elevated intraspecific variation suggesting that it may be under pressure to maintain and/or generate diversity in response to fluctuating insect herbivory pressures.

**Conclusion:**

These data show that intraspecific variation in gene expression is facilitated by an interaction of gene duplication and biological activity. Further, this plays a role in controlling diversity of plant metabolism.

## Introduction

Most eukaryotic genomes have undergone whole genome duplications during their evolutionary history with angiosperms having a particular enrichment in this process [1]–[4]. In addition to whole genome events, small local events can generate tandem duplicated genes which are often considered younger than segmental duplicated genes obtained from a whole genome duplication [1], [5]. Following either tandem or segmental duplication events, one of the duplicated genes can be rendered non-functional via the accumulation of deleterious mutations [6]. Alternatively, both duplicated genes can be maintained if the presence of both copies is advantageous. Over time, the function of these duplicated genes can diverge from the ancestral gene or from each other. This divergence can occur via sub-functionalization such that the duplicate copies obtain differential expression patterns in terms of tissue specificity or stress response [7]. Alternatively, one of the duplicates can obtain a novel function, a process known as neo-functionalization. In a number of species, recent work has associated gene duplication with divergent gene expression patterns in response to developmental or abiotic stress cues [8]–[12].

Gene duplication may also allow partitioning of function among genotypes within a species, leading to genetic differentiation/intraspecific variation. This process is contingent upon genetic neo-functionalization and would suggest that duplicated genes should show higher levels of intraspecific variation in gene expression than “unique” genes. Preliminary evidence supporting the hypothesis that duplicated genes show higher levels of intraspecific variation was obtained in a study with yeast and fruit flies [13]. Given that segmental duplications are typically older than speciation events, it is likely that segmentally duplicated genes have fixed different functions and are in essence acting as unique genes. As such, it would be expected that increases in intraspecific variation may only be detected among younger tandem duplicated genes. In *Arabidopsis thaliana*, several cloned QTL associate intraspecific variation in gene expression with tandem duplication [14], [15]. Yet to date there is little information on the genomic role of gene duplication in controlling intraspecific genetic variation in gene expression or other traits.

Another factor controlling the divergence and maintenance of gene duplicates is the biological function of the gene [5], [9], [16]. It is commonly held that some essential biological processes, such as primary energy metabolism, may be constrained in their ability to vary [17], [18]. However, current studies typically use overarching functional assignments such as “nucleotide binding” or “hydrolase activity” provided by the Gene Ontology consortium. While providing a broad genomic perspective, these categories may obscure the influence of more specific biological function on genetic constraint [19] suggesting the need for a more specific biological definition.

A rich source for testing the relationship between biological function, gene duplication and intraspecific genetic variation are databases describing the metabolic pathways known and predicted within a host of organisms [20], [21]. These databases provide specific metabolic roles for genes acting as a highly refined function prediction. For instance, the Arabidopsis metabolic networks includes genes involved in major energetic and carbon flux pathways that are essential for each cells survival, such as photosynthesis, the calvin cycle, and the TCA cycle (AraCyc; www.arabidopsis.org/biocyc/index.jsp). The critical function of the genes in these primary metabolic pathways may be constrained in the range of gene expression values that the pathways transcripts can occupy and the more specific definition of their biological activity may enhance our ability to detect this.

In contrast to primary metabolism, genes involved in secondary metabolite pathways are marked by a high level of inter and intraspecific variation [22]–[24]. The evolution of this diversity is predicted to be driven by gene duplication and consequent neo-functionalization of enzymes [15], [25]–[27]. Glucosinolates are sulphur-rich, amino acid-derived compounds that form a major class of secondary metabolites in Arabidopsis. These compounds are hydrolyzed by an endogenous thioglucosidase, myrosinase, releasing toxic products upon disruption of plant cells by harvesting, processing, or mastication [28]. Aliphatic glucosinolates are subject to diverse selection regimes in the wild [27], [29], [30]. We hypothesize that such secondary metabolite pathways may have higher levels of gene duplication and greater gene expression diversity than primary metabolite enzymes. Thus, Arabidopsis metabolism provides an excellent model to study how gene duplication and biological function interplay to control constraint, sub-functionalization and/or neo-functionalization following gene duplication.

In this study, I investigated the role that gene duplication and metabolic pathway organization play in controlling gene expression variation within *Arabidopsis thaliana* natural accessions. I also measured gene expression variation in developmental and abiotic stress datasets to allow comparison with other publications focused on gene duplication and divergence of response patterns. Recent tandem and older segmental duplicated genes showed a significant increase in intraspecific variation of gene expression in comparison the average gene, while unique genes showed lower levels of intraspecific variation. Gene expression variation of segmental and tandem duplicated genes was controlled by a greater number of genetic loci, and these loci had larger effects on gene expression variation for tandem duplicated genes. Gene participation in specific metabolic pathways predicted the level of intraspecific variation in gene expression, with major energy and amino acid pathways having relatively lower levels of gene expression variation. This suggests that these primary metabolism pathways are genetically constrained. In contrast, a major secondary metabolite pathway in Arabidopsis showed a significantly increased level of gene duplication and intraspecific gene expression variation, suggesting that this pathway may be structured to cope with fluctuating selection in the wild. As such, the influence of gene duplication on intraspecific gene expression variation partly depends upon the biological function of the gene involved.

## Results

### Duplicated Genes have more variable transcript accumulation

To understand the role that gene duplication and gene expression diversity may play in controlling metabolic pathways, I analyzed transcript accumulation for the complete genome using four Affymetrix ATH1 microarray datasets. The first two datasets are two independent replicated experiments focused on natural genetic variation across multiple Arabidopsis accessions [Accessions I; [31] and Accessions II;[32]]. These provide a measure of intraspecific gene expression variation. The additional datasets are replicated experiments measuring alteration of the transcriptome through response variation within a single genotype [Development; [33]) and by abiotic stress (Abiotic; [34]]. These provide a comparison with other studies focused on gene duplication and the evolution of divergent gene expression patterns in response to development or abiotic stress in a single genotype [5], [9], [16].

For each replicated experiment within a dataset, the mean transcript value per experiment was calculated. To compare transcriptomic responses between datasets, two related indicators of transcript variance were estimated per transcript per dataset, the variance mean ratio (VMR) and the coefficient-of-variance (CV) [35], [36]. Given that the CV and VMR were obtained using the mean transcript level per experiment, they act as estimates of transcript variation in response to perturbations in development, abiotic stress or natural genetic variation rather then simply a measure of experimental variation. To control for errors introduced from cross-hybridization, only genes with unique probes sets were utilized for the analysis. All probe-sets annotated as recognizing multiple genes were removed. Both the VMR and CV showed that the average Arabidopsis transcript was most sensitive to developmental perturbation (Tables 1 and 2). Interestingly, both studies querying genetic variation showed similar levels of transcript variance, albeit lower levels than calculated for the response datasets (Tables 1 and 2).

**Table 1:** **VMR for genes with different duplication states.** Shown is the average VMR for the whole genome, unique genes, segmental duplicated genes and tandem duplicated genes.

Dataset	Unique	Genome	Segmental Duplicate	Tandem Duplicate
Accession I	0.027	0.030	0.031	0.054
Accession II	0.020	0.022	0.027	0.039
Developmental	0.201	0.235	0.301	0.421
Abiotic	0.045	0.051	0.063	0.085

**Table 2:** **CV for genes with different duplication states.** Shown is the average CV for the whole genome, unique genes, segmental duplicated genes and tandem duplicated genes.

Dataset	Unique	Genome	Segmental Duplicate	Tandem Duplicate
Accession I	0.052	0.056	0.054	0.078
Accession II	0.042	0.044	0.049	0.058
Developmental	0.161	0.174	0.201	0.256
Abiotic	0.068	0.073	0.081	0.096

**Figure 1:**
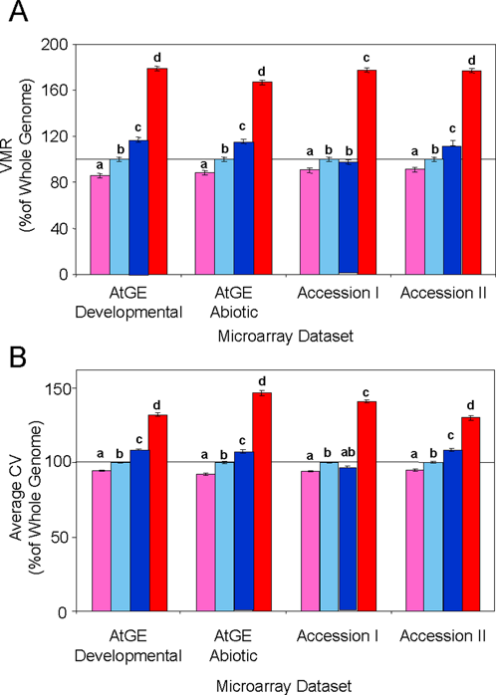
Gene duplication status alters transcript variance. Genes within each of four independent microarray datasets were grouped as unique (pink), segmental duplicated (dark blue) and/or tandem duplicated (red) and the average of two measures of transcript variance was determined across all genes within these groups as well as for the whole genome (light blue). Within each dataset, letters distinguish gene groups that differ significantly at *P*<0.001 as determined by bootstrapping. Within each dataset, variance estimates were standardized to the whole genomic average (set to 100%) indicated by the horizontal line. VMR: variance mean ratio. CV: the coefficient-of-variance. A. Average VMR for the transcripts. B. Average CV for the transcripts.

Gene duplication has been associated with enabling neo- and sub-functionalization whereby duplicate genes may diverge [7], [8], [12], [37]. To test if gene duplication is associated with increased transcript variation, I used a previous study that classified genes within the Arabidopsis genome as unique, segmental duplicate or tandem duplicate [5]. In this study, segmental duplicated genes contained both transpositional duplicates and polyploidy orthologs. In all four datasets, transcripts associated with tandem duplicated genes showed dramatically increased variance across perturbations and genotypes in comparison to the whole genome (Figure 1). Additionally, segmental duplicated genes showed elevated variation for three of the four datasets and unique genes had lower than expected variance across the response and natural genetic variation datasets (Figure 1). This shows that tandem duplications allow for increased variation in gene expression within Arabidopsis. At least one of two datasets suggests that the same is true for segmental duplications (Figure 1). As such, subdivision of gene function across duplicates may manifest as differential expression patterns across multiple genotypes or as differential expression patterns within a single genotype.

Previous studies have shown that gene duplication or polymorphism occur disproportionately within specific gene families and Gene Ontology (GO) classes [5], [19], [38]. As expected, all four datasets showed increased transcript variation for specific GO terms associated with elevated gene duplication, such as ‘Response to abiotic and biotic stimulus’ [5], as well as for gene families showing increased sequence polymorphism, e.g. Cytochromes P450, NBS-LRR and F-box genes [38] (Kliebenstein, unpublished data). However, neither gene family nor GO term is a precise measure of biological function.

### Comparison of Accession Variation with Abiotic Variation

**Figure 2 pone-0001838-g002:**
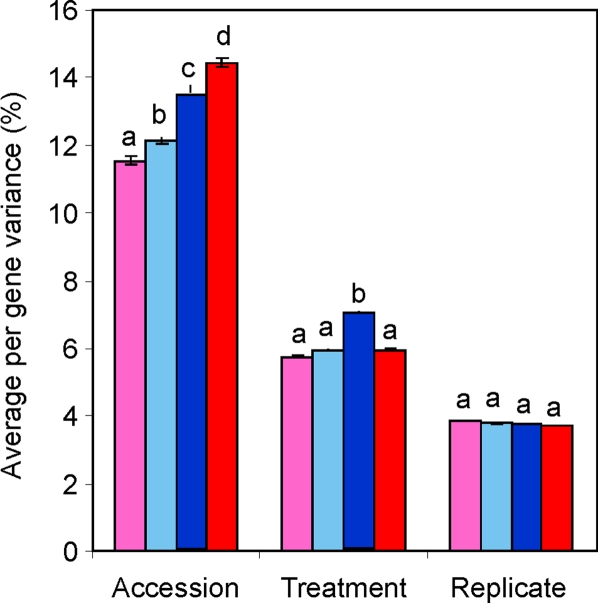
Partitioning of transcript variance in a factorial analysis of genetic and abiotic variation. Individual transcripts' variance components were partitioned via ANOVA within a replicated factorial experiment comparing natural genetic variation with an abiotic stress treatment. The average percent of total variation per individual transcript is shown for unique genes (pink), the whole genome average (light blue), segmental duplicated genes (dark blue) and tandem duplicated genes (red). Within a factor, letters show significantly different duplication classes.

To directly compare the level of transcript variance due to natural genetic variation versus an abiotic treatment, I obtained a factorial dataset in which transcript accumulation between two Arabidopsis accessions, Bay-0 and Shahdara, was compared in the presence or absence of exogenous salicylic acid in two replicate experiments [39]. We calculated the total variance for each transcript across all microarrays and estimated the percent of per transcript variance that was due to the main experimental variables (accession, treatment and replicate) as well as their interaction terms. This showed that differences between the two accessions was a greater source of gene expression variance than treatment effect (Figure 2)[40]. This was not due to polymorphisms that impact hybridization as there was no difference in genetic variance estimates for genes with or without a detected SFP as previously observed with these datasets [31], [39], [41]. In this analysis, both segmental and tandem duplicate genes had a significant enhancement in per transcript variance due to accession when compared to the average transcript (Figure 2). Conversely, unique genes had a diminished level of transcript variance. Interestingly, only segmental duplicate genes had an altered level of transcript variance in response to the treatment variation (Figure 2). Tandem and segmental duplicate genes did not have elevated transcript variance due to the replicate suggesting that tandem or segmental duplicated genes are not noisier than unique genes (Figure 2). This analysis further suggests that both segmental and tandem duplicate genes have more intraspecific gene expression variation than the average gene in the Arabidopsis genome.

**Figure 3:**
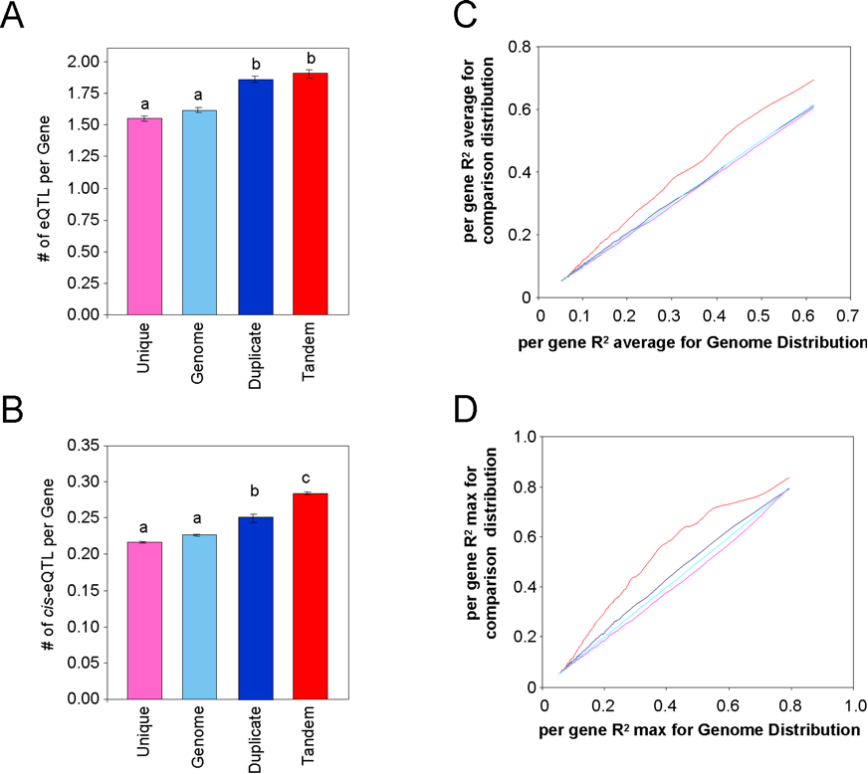
Impact of gene duplication on eQTL analysis. An analysis of eQTL detected within 211 replicated Bay-0×Sha recombinant inbred lines was used to address the impact of gene duplication on genetic control of transcript variation. For all graphs, pink shows unique genes, light blue is the genome average, dark blue are the segmental duplicated genes and red is the tandem duplicated genes. A. Average number of *cis*-eQTL per transcript per duplication class. Letters show duplication classes with statistically significant differences. B. Average number of *cis*-eQTL per transcript per duplication class. Letters show duplication classes with statistically significant differences. C. QQ plot comparing distributions of the average eQTL R^2^ for each transcript among duplication classes. The X-axis shows the average eQTL R^2^ for transcripts every percentile within the whole genome. The Y-axis shows the average eQTL R^2^ for transcripts every percentile within the comparison transcript set. D. QQ plot comparing distributions of the maximum (max) eQTL R^2^ for each transcript among duplication classes. The X-axis shows the max eQTL R^2^ for transcripts every percentile within the whole genome. The Y-axis shows the max eQTL R^2^ for transcripts every percentile within the comparison transcript set.

To provide another measure of intraspecific gene expression variation, I obtained a large replicated dataset investigating quantitative trait loci controlling gene expression (eQTL) between the Bay-0 and Sha accessions. If gene duplication allows for an increase in variation between accessions, then duplicated genes should have a higher frequency of genetic polymorphisms controlling their transcript abundance. In agreement with this prediction, duplicated genes had on average more total eQTL per transcript than the unique genes or the whole genome average (Figure 3A). This was partially due to duplicated genes, both tandem and segmental, more frequently having *cis-*eQTLs (eQTLs located at their physical positions) (Figure 3B). Interestingly, eQTLs detected for tandem duplicate genes have larger allelic effects than eQTLs for segmental duplicate genes (Figure 3C and D). This suggests that duplicated genes are less constrained in their ability to accumulate genetic polymorphisms influencing transcript accumulation than the average Arabidopsis gene.

### Metabolic Consequences of Transcript Variation

Gene duplication is thought to provide much of the genetic material allowing plants to generate a vast diversity of metabolites. To test how gene duplication and associated gene expression variation may shape Arabidopsis metabolism, I focused on transcripts associated with specific metabolic pathways using the AraCyc databases predictions [20], [21]. I then measured the average transcript variance (CV and VMR) across the transcripts within a metabolic pathway to develop a pathway estimate of variance and utilized bootstrapping to generate an empirical distribution of transcript variance. This empirical distribution was used to test for a difference between the specific metabolic pathways CV and the expected CV for a similarly-sized group of genes randomly drawn from the genome.

**Figure 4:**
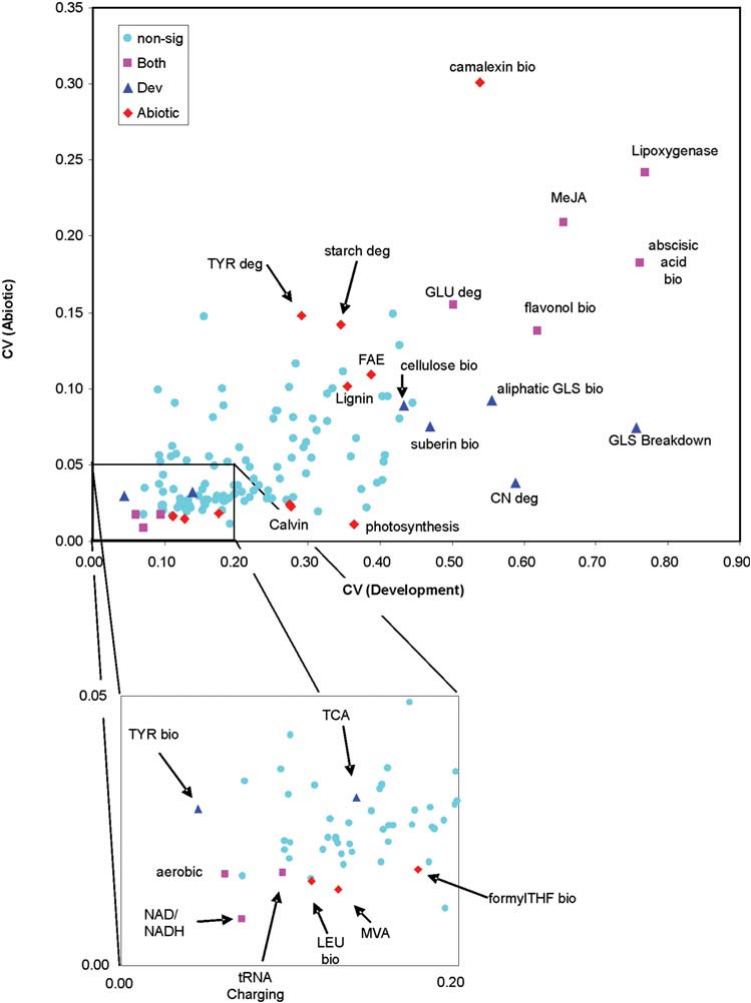
Comparison of average metabolic pathway CVs between development and abiotic stress datasets. The average CV per metabolic pathway for the Development and Abiotic microarray studies are plotted with the significance of each spot within the two studies represented by both color and shape: Pink squares = pathways with a significant deviation from the whole genome in both studies; red diamonds = deviation from the whole genome in Abiotic only; dark blue diamonds = deviation from the whole genome in Developmental only; light blue circles = no observed deviation from the whole genome average. The inset expands the lower left part of the graph. Significantly differing pathways are annotated to indicate biosynthesis (‘bio’) or degradation (‘deg’). GLS stands for glucosinolate. FAE stands for fatty acid elongation. CN stands for cyanate.

To test the utility of pathway CV estimates for interpreting metabolic pathway response to experimental variation, I investigated the response datasets. This identified a significant positive correlation between metabolic pathway transcript CVs in response to developmental and abiotic perturbations (P <0.001, R^2^ = 0.43 Figure 4). This is not unexpected given that both datasets utilize a single Arabidopsis genotype, Col-0, and similar signaling pathways are involved controlling gene expression in response to development and abiotic stress. The major energy conversion and amino acid biosynthetic pathways (e.g. TCA cycle, aerobic respiration, etc) showed significantly diminished pathway transcript CV in both datasets as would be expected for these essential metabolic pathways (Figure 4). Interestingly, pathways for two hormones responsible for controlling differential plant development and abiotic stress responses, abscisic acid and jasmonic acid, showed a significant increase in transcript CV within both datasets (Figure 4)[42]–[46]. The Arabidopsis secondary metabolite pathways further support the relationship between pathway transcript CV and biological function. A pathogen inducible compound, camalexin, only shows a significantly enhanced transcript CV in the Abiotic dataset. In contrast, the more developmentally controlled glucosinolate pathways show significantly enhanced transcript CV in only the Developmental dataset (Figure 4) [47]–[51]. Thus, the pathway level transcript CV estimate identifies predicted metabolic pathway responses within the response datasets.

### Metabolic Pathways and Natural Genetic Variation

**Figure 5:**
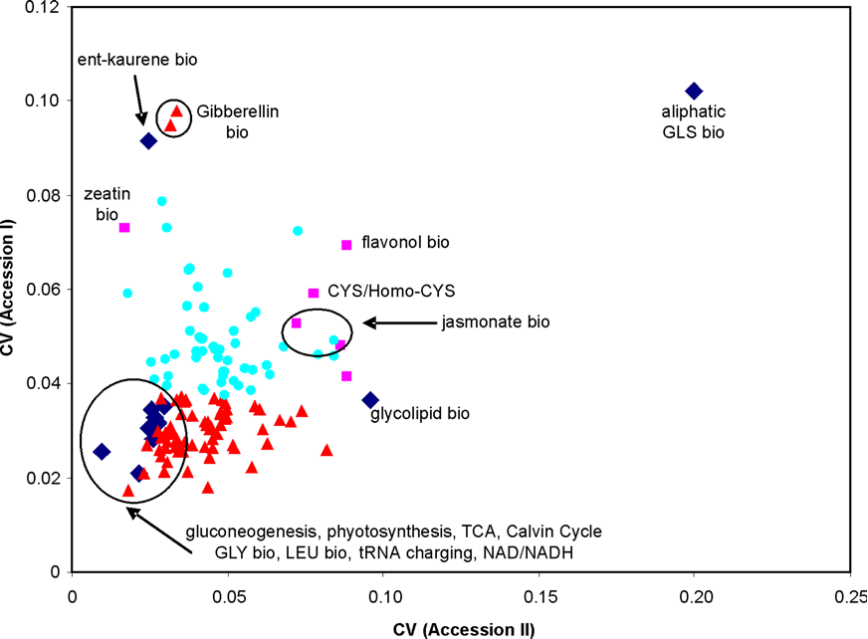
Comparison of average metabolic pathway CV between two independent genetic variation studies. The average CV per metabolic pathway for Accession I and Accession II are plotted with the significance of each spot within the two studies represented by both color and shape: black diamonds = pathways with a significant deviation from the whole genome in both datasets; red triangles = deviation from the whole genome in only Accession I; pink squares = deviation from the whole genome in only Accession II; blue circles = no observed deviation from the whole genome average. Significantly differing pathways are annotated to indicate biosynthesis (‘bio’) or degradation (‘deg’). Amino acid pathways are represented by their capitalized three letter code. GLS stands for glucosinolate.

**Figure 6:**
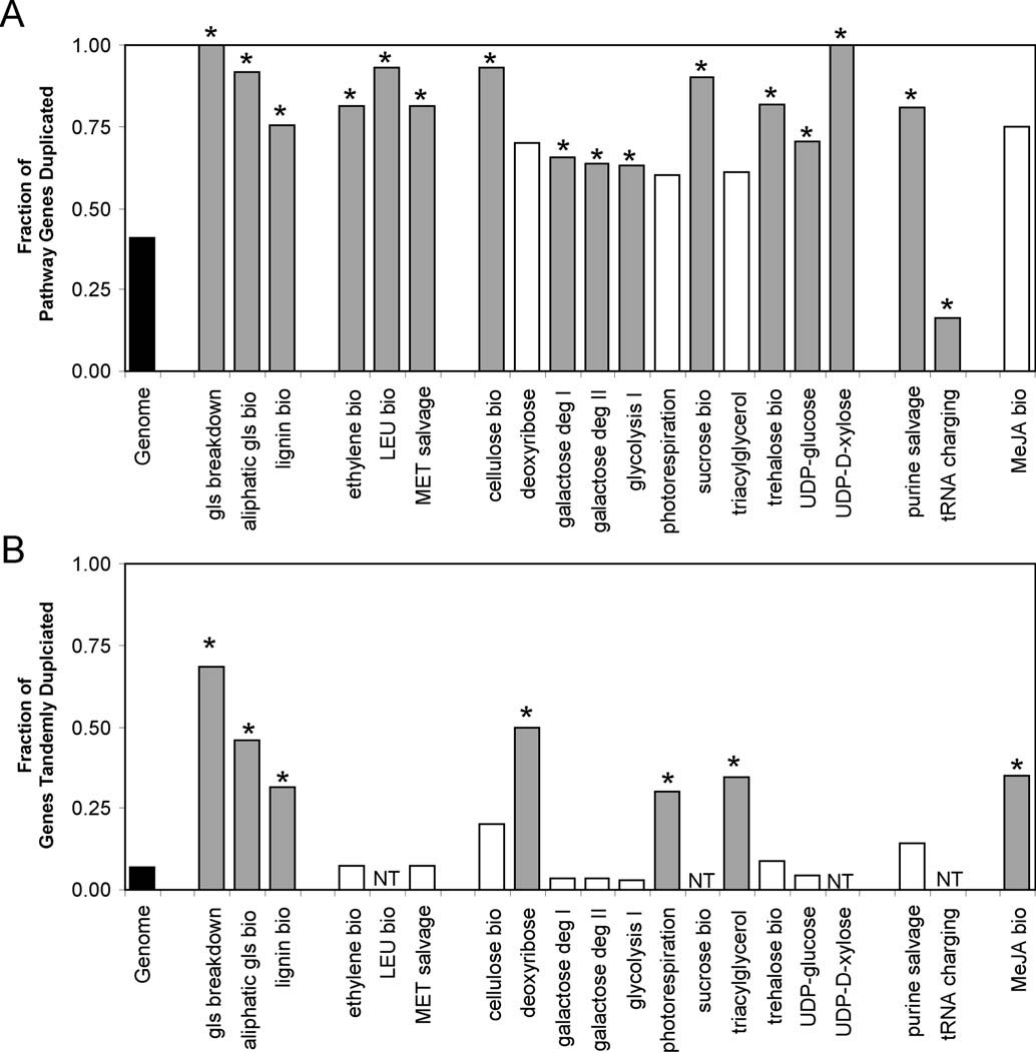
Biased frequency of gene duplication in metabolic pathways. The level of gene duplication within metabolic pathways that show biased gene duplication frequency is presented. χ^2^ analysis was used to test duplication frequency within each metabolic pathway for deviation from the whole genome expectation. * and blue bars show pathways that have a statistically significant bias in gene duplication after adjusting for multiple comparisons at an FDR of 0.05. The bars are separated into biochemical groups (left to right: secondary metabolism, amino acid related, energy related, nucleotide related and hormone related). A. Total duplicated genes per metabolic pathway. B. Tandem duplicated genes per metabolic pathway.

I next utilized the pathway level transcript CV to compare intraspecific variation measured in the two Accession datasets. A low pathway level transcript CV would suggest genetic constraints limiting gene expression diversity of pathway members. In contrast, elevated levels of pathway transcript CV may result from selection for increased gene expression diversity. The availability of two independent ATH1 microarray datasets investigating natural genetic variation in transcript accumulation within Arabidopsis allows a replicated analysis of metabolic pathways to detect biased transcript variance. Major energy and amino acid pathways showed significantly diminished pathway transcript variance in comparison to the average random gene set in both independent accession datasets (Figure 5) suggesting that transcript variance in these essential biochemical pathways is genetically constrained. The photosynthesis and calvin cycle pathways do show significant pathway variance within the development dataset displaying the potential of these pathways to vary (Figures 4 and 5). This increased genetic constraint in comparison to the average metabolic pathway may relate to a relative lack of gene duplicates in these pathways. For instance, the tRNA charging pathways have a paucity of gene duplicates in comparison to the random expectation (Figure 6 and Table S4). Overall, the number of tandem duplicates within a pathway was positively correlated to the pathway's average transcript CV within the Accession datasets (P<0.001, R^2^ = 0.21, N  = 135; for this test, the Aliphatic Glucosinolate Biosynthetic pathway was removed given its high CV). This suggests that gene duplication status for the different metabolic pathways can predict the level of genetic variation for gene expression within a given metabolic pathway. Interestingly, none of the pathways with either elevated or diminished pathway level transcript CV showed significantly different estimates of sequence polymorphism in comparison to randomly generated pathways (Table S5)[52].

**Figure 7:**
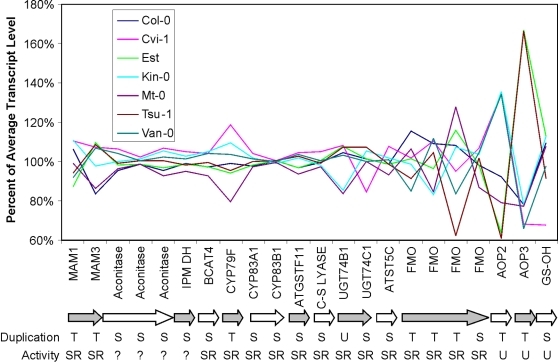
Variation of transcripts within the aliphatic glucosinolate biosynthetic pathway. The variation of each aliphatic glucosinolate biosynthetic transcript across seven Arabidopsis accessions from the Accession I dataset is plotted. Accumulation of each transcript within each accession was standardized to the average transcript level across the accessions. Accession coding is shown in the legend. Genes for the different transcripts are ordered from first to last (left to right) biosynthetic step. Arrows group genes for different biosynthetic steps. ‘Duplication’ indicates whether each gene was classified as being a tandem (T), segmental (S) or unique (U) gene [5]. ‘Activity’ shows whether each gene has a unique (U), redundant (R), semi-redundant (SR) or unvalidated (?) activity. Activity was defined via analysis of published literature such that if a loss of the gene abolished the biosynthetic reaction it was classified as unique, if the loss partially abolished the reaction it was classified as semi-redundant, and if gene loss had no effect on the reaction it was deemed redundant. A question mark means that gene loss has not yet been evaluated. As there is only one probeset on the ATH1 microarray for *CYP79F1* and *CYP79F2*, they are jointly annotated as CYP79F in this study.

In contrast to the constrained major energy metabolism pathways, the aliphatic glucosinolate biosynthetic pathway showed significantly elevated pathway level transcript CV in both accession experiments suggesting that the transcripts in this pathway may be under selection for increased diversity. This secondary metabolite pathway is a major insect and pathogen defense pathway within Arabidopsis [53]–[55]. In agreement with the increased transcript level variation, significant genetic variation for both content and structure of aliphatic glucosinolates is potentially influenced by diverse selective pressures [27], [56], [57]. While it is possible that this increased pathway level transcript CV could be caused by natural genetic variation in one or two transcription factors, accessions did not significantly covary for the transcripts in the aliphatic glucosinolate biosynthesis pathway (Figure 7). Interestingly, transcript CV increases along the pathway and is highest for transcripts determining structural variation in the final aliphatic glucosinolate, e.g. MAMs, CYP79Fs, FMOs and AOPs [14], [15], [58]–[61]. The increased transcript CV for the aliphatic glucosinolate pathway is associated with significantly elevated levels of gene duplication for the genes within this pathway (Figure 5 and 6).

Several metabolic pathways showed differential levels of transcript variation between the two Accession datasets. This is most striking for the ent-kaurene biosynthetic pathway that has significantly elevated pathway transcript variance in Accession I but significantly diminished transcript variance in Accession II (Figure 5). Ent-kaurene is a precursor for gibberellin biosynthesis, whose pathways show a similar pattern of variation (Figure 5). Gibberellic acid is an important developmental regulator that is also believed to play a role in controlling flowering time under short day conditions [62]–[64]. The plants for the Accession I dataset were grown under short-days and harvested just prior to flowering onset for some of the accessions. In contrast, the plants for the Accession II dataset were grown under long-days and harvested long before flowering. As such, the Accession I dataset may identify genetic variance in gibberellic acid associated with flowering time, a trait for which variation is likely under selection [65]–[67]. In contrast, the experimental conditions for Accession II may have accentuated the constrained developmental roles of gibberellic acid in seedlings, decreasing detection of genetic variation in transcript accumulation. Future work will be required to better understand the role of genotype × environment interactions in controlling the measurement of genetic variation in transcript accumulation.

## Discussion

### Tandem Gene Duplication and Genetic Constraint

This study explores how gene duplication and metabolic function interact to control intraspecific variation in gene expression. Tandem duplicated genes in *Arabidopsis thaliana* show increased levels of intraspecific gene expression in all tested datasets (Figures 1–3 and Tables 1 and 2). This includes two different samples of Arabidopsis accessions, a detailed comparison of abiotic and genetic variation within two accessions, and the frequency of transcript accumulation polymorphisms that cause eQTL. Tandem duplications are likely younger than segmentally duplicated chromosome regions, which were probably generated during whole genome duplication events approximately 20–40 million years ago [1], [2], [68]. As such, the function of segmental duplicated genes is more likely to have been fixed than the tandem genes. In agreement with this hypothesis, the two Accession datasets showed that tandem duplications showed significantly higher intraspecific gene expression variation than the segmental duplicated genes (Figure 1 and 2). However, the segmental duplicates showed enhanced intraspecific gene expression variation in comparison to unique genes in both accession datasets as well as in the eQTL analysis. While the number of eQTLs did not differentiate between the duplication classes, the distribution of effect sizes showed that tandem duplicates typically had larger effect eQTLs (Figure 3). Interestingly, both duplication classes had more *cis* and *trans*-eQTLs than the average gene. The increased level of trans-eQTLs suggests that duplicated genes are involved in less constrained pathways than unique genes, but this remains to be further investigated. Overall, these data suggests that both tandem and segmental duplicate genes have greater potential to facilitate the generation of intraspecific variation than unique genes, but the tandem genes make a greater contribution.

While we eliminated entire probe-set cross-hybridization by not analyzing these probe sets, any probe level cross-hybridization between duplicated genes may remain. However, if a probe hybridizes to two or more transcripts its effect will be limited in two ways. First, the other 10 probes in a probe set will still provide an accurate estimate. Secondly, any probe that binds two or more transcripts will be averaging across transcripts and likely lead to decreased estimates of variance. Previous work with the eQTL population or accession data suggested that any relationship between probe level variation and transcript level variation while significant was minimal in comparison to the probe sets variation [31], [39]. Together, this suggests that individual probes are not likely a significant contribution to our variables and that our estimates are conservative.

### Metabolic Pathways and Gene Expression Constraint

Essential metabolic pathways required for the production and movement of energy within the plant showed significantly lower levels of gene expression variation across development, abiotic stress and natural genetic variation (Figures 4 and 5). This suggests that gene expression for these pathways is constrained across a range of external, internal and genetic stimuli. This constraint agrees with the essentiality of these pathways, e.g. aerobic respiration and the TCA cycle, to an individual cells survival. Only one of these less variable pathways (tRNA charging) had a lower than average level of gene duplication, suggesting that the constraints on these pathways are not associated with altered duplication patterns. The two exceptions to the observation that major energy metabolic pathways are constrained are the calvin cycle and photosynthesis pathways. These pathways showed significant genetic constraint but no statistical support for developmental constraints most likely due to the difference in photosynthetic capacity between roots and shoots. The observed genetic constraints for these two pathways are not due to a technical issue limiting the ability to accurately estimate CV (Figure 5).

Development and abiotic stress are sometimes considered a comparison of internal versus external cues regulating gene expression variation. Interestingly, our analysis of metabolic pathways identified a significant positive correlation in pathway variation between the development and abiotic stress datasets (Figure 4). This positive relationship between development and abiotic stress suggests an association between gene expression responses to external and internal cues. This association is supported by a host of observations wherein plant responses to external factors are regulated via plant hormones that also control internal developmental cues [42]–[46], [69]. Exploring pleiotropic effects of similar signaling compounds being used for both responses to internal (development) and external (abiotic stress) cues upon gene expression divergence will be an interesting avenue for future research.

### The Aliphatic Glucosinolate Biosynthetic Pathway and Sub-functionalization versus Neo-functionalization

Within the aliphatic glucosinolate biosynthetic pathway, increased transcript variation across naturally variable Arabidopsis accessions is associated with tandemly duplicated enzymes. This tempts the conclusion that these gene families are undergoing genetic sub-functionalization. However, biochemical analysis of these gene families shows that they have already undergone neo-functionalization, creating new biosynthetic activities in the duplicate enzymes. Examples include *MAM1* v *MAM3* [58], [70], *CYP79F1* v *CYP79F2* [60] and *AOP2* v *AOP3* [15]. As such, variance in gene expression leads to dramatic variation in glucosinolate structure and serves as evidence of genetic and biochemical neo-functionalization [56], [71], [72]. Even for those enzymatic steps where the tandem duplicates appear to have similar biochemical properties, manipulation of individual genes has a measurable consequence for the resulting glucosinolate output [59], [73], [74]. Visible phenotypic changes associated with changes in expression of these loci indicate that these loci are not completely redundant. As such, the aliphatic glucosinolate biosynthetic pathway illustrates the difficulty in genomic/bioinformatic analysis of neo- versus sub-functionalization. Expression analysis would argue that sub-functionalization of the tandem duplicates has occurred, but biochemistry reveals neo-functionalization of the tandem duplicates.

### The Aliphatic Glucosinolate Biosynthetic Pathway and Intraspecific Variation

Pathway level transcript variation for the aliphatic glucosinolate biosynthetic pathway was a dramatic outlier in both accession datasets (Figure 5 and Table S3). Gene expression variation across the different accessions was distributed such that the enzymatic steps responsible for determining the final chemical structure had elevated gene expression variation, suggesting that variation in this pathway may be selected to generate structural diversity (Figure 7). However two of these enzymatic loci responsible for structural diversity, *MAM1/3* and *AOP2*, also pleiotropically regulate gene expression for the whole biosynthetic pathway, thus complicating our ability to separate the effects of content versus structure in this experiment [75]. A potential explanation for increased genetic diversity in the aliphatic glucosinolate biosynthetic pathway may be that gene expression diversity enables response to fluctuations in natural insect populations between different generations. Aliphatic glucosinolates provide a positive fitness benefit in the presence of generalist herbivores but can impart both a cost of production in the absence of herbivores and a fitness cost in the presence of specialist herbivores [29], [76], [77]. As such, natural fluctuations in insect herbivore populations would lead to fluctuating selection pressures on aliphatic glucosinolates within Arabidopsis. Thus, Arabidopsis may respond to these unpredictable fluctuations via standing genetic variation in this pathway [56]. Similarly, evidence for the contribution of gene duplication and intraspecific variation to defense mechanisms has been found in studies of plant gene-for-gene resistance and cone snail toxin [78], [79].

### Conclusion

This work shows that the availability of gene duplications and the function of biochemical pathways interact to influence gene expression variation across a diverse array of biological and genetic factors. As such, I suggest a model in which gene duplication provides raw material for evolution but, at least in metabolic pathways, the biology of the pathway determines the likelihood that duplicated genes are maintained and evolve altered functionality. In secondary metabolic pathways where variation is required, duplicates will be maintained and evolve diverse expression patterns. In primary metabolic pathways where variation may be detrimental, this may actually select against the presence of duplicated genes and variable expression patterns. In the absence of functional biochemical evidence, it is difficult to classify gene expression changes between tandem duplicates as true sub-functionalization versus neo-functionalization coupled with intraspecific gene expression variation. Future tests may reveal whether the variation in metabolic pathways showing elevated transcript CV is an essential component of Arabidopsis fitness.

## Materials and Methods

### Microarray data

Four previously published datasets encompassing a large number of transcriptomic analysis utilizing Arabidopsis Affymetrix ATH1 microarrays were used for this experiment. One dataset, hereafter referred to as Development, contains replicated analysis of transcript variation across a variety of developmental stages and tissue types from *Arabidopsis thaliana* [33]. A second dataset, hereafter annotated as Abiotic, contains replicated analysis of *Arabidopsis thaliana* transcriptional response to multiple abiotic stresses [34]. Two independent datasets are from replicated experiments querying natural variation in gene expression across Arabidopsis accessions. These are annotated as Accession I, containing seven replicated accessions, Col-0, Cvi-1, Est, Kin-0, Mt-0, Tsu-1 and Van-0 [31], and Accession II containing ten replicated accessions Bay-0, C24, Col-0, Cvi-1, Est, Kin-0, Ler, Nd-1, Shakdara and Van-0 [32]. These accession experiments were independently conducted, with overlap of five accessions. Accession I microarrays measurements used five-week old plants grown under short day conditions (10:14 hours light:dark) [31], while Accessions II analyzed RNA from 12-day old plants grown under long days (16:8 hours light:dark) [32]. The short day conditions can be considered non-inductive for flowering, while long days would induce flowering [63]. All microarrays were quantile normalized via GC-RMA and transcript levels converted to their log_2_ values. The mean log_2_ transcript accumulation per gene per experimental unit (accession, tissue, stress, or time point) was used for further analysis. Only biological replicates were utilized and technical replicates were discarded. When technical replicates were present, one array was chosen at random to represent that sample.

### Transcript variance measures

To compare variance across experiments and transcripts, I first estimated the per transcript variance (σ^2^) across the mean log_2_ transcript accumulation per experimental unit for each transcribed locus measured within a given experimental dataset (Table S1). The mean (µ) for each transcript was determined across all microarrays for the given experimental dataset. Only biological replicates were utilized and technical replicates were discarded. These mean and variance values per dataset were used to generate two standardized measures of transcript accumulation variance comparison of variances for each transcript across datasets: the variance-to-mean ratio (VMR = σ^2^/µ) and the unit less coefficient of variance (CV = σ/µ)[35], [36]( Table S1). These measures were independently estimated for each of the four microarray datasets. Only probe-sets that identified unique genes were utilized in this analysis and all probe-sets that identified multiple genes were discarded to control for potential cross-hybridization. Any cross hybridization at the individual probe level will likely lead to lower variance estimates for both duplicated genes.

### Gene Duplication

A previous analysis of gene duplication within Arabidopsis was used to define genes as either unique (no duplicate copy), segmental duplicated (a duplicate somewhere in the genome but not tandem) and tandem duplicated (the duplicate gene is next to the tested gene) [5]. The high-stringency analysis from this previous work provided a base definition of duplication status for this analysis. Tandem duplicated genes are considered a sub-group of duplicated genes for this analysis (Table S1). This analysis includes 21,460 genes for which there are both microarray data and assigned duplication status. 12,676 genes are classified as unique, defined as having no duplicate at a stringency of >50% identity and >90% alignment length [5]. 7,272 genes are classified as being segmental duplicates and 1,512 genes are classified as being tandem duplicates with the duplicate copies immediately neighboring each other [5].

### Metabolic pathway definition

Aracyc version 3.1 was utilized to define metabolic pathways [20], [21]. Specific secondary metabolite pathways were edited to include recently published enzymes and genetic loci [59], [80]. A previously un-annotated pathway, glucosinolate breakdown, was created using recently published data [53], [54], [81]–[83]( Table S2). Only those metabolic pathways with at least five genes and five separate transcripts measured by the ATH1 microarray were further analyzed. Biochemical activity of genes involved in the aliphatic glucosinolate biosynthetic pathway was defined from the literature and unpublished data for three FMO genes [15], [58]–[60], [70], [73], [74], [84]–[87].

### Duplicated Gene Variance

For the four independent datasets, VMR and CV were independently determined for all transcripts (Figure 1). The genes were then separated by duplication status and the mean VMR and CV were determined for unique, segmental duplicated and tandem duplicated genes for each dataset. To test if the mean VMR and CV for these duplication groupings were significantly different from the genomic mean, a bootstrap analysis was conducted. Random samples containing an identical number of genes to each of the three duplication groupings (12,767 genes for the unique, 7,272 genes for the segmental duplicate and 1,512 for the tandem duplicate group (Figure 1)) were drawn from the full genome and the mean VMR and CV of each of these samples was determined. Repeated 2,000 times, this generated a random distribution of mean VMR and CV values for each of the four microarray datasets. Independently for each microarray dataset, the mean VMR and CV for the unique, segmental duplicated and tandem duplicated genes were then compared to the appropriate random sampling distribution to test for a significant difference from the genomic mean. To test the significance of differences between the segmental and tandem duplicated gene groups for each dataset, a bootstrapping analysis was conducted such that 8,784 genes were randomly picked from the whole genome, with 1,512 of these genes being randomly assigned as tandem duplicates and the other 7,272 genes being assigned as segmental duplicates. The average CV and VMR of these two groups was determined, the difference in values obtained, and this process repeated 2,000 times. The difference between the observed values of CV and VMR for the duplicated and tandem duplicated genes for each dataset was compared to this random distribution to test if the observed difference was significantly different from a random expectation.

### Analysis of Accession Variance Components

A microarray dataset comparing the response of seven Arabidopsis accessions, Col-0, Cvi-1, Est, Kin-0, Mt-0, Tsu-1 and Van-0, to exogenous salicylic acid treatment was utilized to directly assess the impact of gene duplication on different sources of gene expression variation [40]. I used a mixed linear model ANOVA in SAS to analyze the GCRMA normalized log_2_ transcript level (gene expression) data from the factorial experiment to estimate the variance contributions of accession versus treatment. For each gene, the transcript level of Accession *g* under SA treatment *j* for the replication *r* is denoted as *y_gnjkr_*. The ANOVA model for the log_2_-transformed expression levels is: *log_2_(y_gjr_) = µ+S_j_*+*G_g_*+*R_r_*+*SG_gj_+SR_jr_+GR_gr_+ε_gjr_* where *g* = 1, …,7; *j = *1,2; and *r* = 1, 2, 3. The main effects are denoted as *G*, *S*, and *R* and represent gene, treatment, and replicate respectively. Replicate was treated as a main effect to estimate its impact on transcript variance within this specific experiment. The error, *ε_gikr_*, is assumed to be normally distributed with mean 0 and variance σ_ε_^2^. The σ^2^ for each main effect and interaction term was divided by the total σ^2^ for each gene to obtain the percent of variance per term. This was repeated for all genes and for each term, producing an average percent variance for the four different gene groups, unique, whole genome, duplicate and tandem duplicate. The significance of deviation from the genome average for the unique, segmental duplicate and tandem duplicate groups was estimated via bootstrap analysis as described above.

### Analysis of eQTL Bias

The expression QTL (eQTL) position and R^2^ for each eQTL for each transcript was obtained from a previous eQTL mapping experiment comparing two Arabidopsis accessions, Bay-0 (Bayreuth) and Sha (Shahdara) [39]. For all transcripts, the average number of eQTL, the frequency of a *cis*-eQTL, the average R^2^ per eQTL, and the maximum R^2^ per eQTL was determined. The genome average for each variable was determined and then re-measured for each duplication group. The significance of deviation from the genome average for the unique, segmental duplicate and tandem duplicate groups was estimated via bootstrapping as described above.

### Metabolic Pathway Variance

For the four independent datasets, the mean VMR and CV were independently determined for all transcripts included in a given metabolic pathway (Table S3). Bootstrapping analyses were employed to test if the mean VMR and CV for transcript accumulation within each metabolic pathway were significantly different from a random genomic sample. For each round of the bootstrap, 5, 10, 15, 20, 25, 30, 35, 40, 45 and 50 genes were drawn at random from the given dataset and the mean VMR and CV determined across these genes. These gene numbers represent random metabolic pathways containing different numbers of genes. This was repeated 2,000 times to generate a random distribution of VMR and CV for each dataset. This generated 40 different random sampling distributions (four datasets×10 pathway sizes). For each metabolic pathway, the number of genes, N, was rounded to the nearest five and the random distribution from the appropriate dataset and pathway size utilized to test if that pathways VMR or CV differed significantly from a random “pathway” of similar size. *P* values were tested for significance under an FDR of 0.05 (Table S3).

### Metabolic Pathway Duplication

For each metabolic pathway, the number of segmental duplicated and tandem duplicated genes was determined from previous analysis (Tables S1 and S4) [5]. The genomic frequency of segmental duplicated and tandem duplicated genes was then used to generate the expected numbers of segmental duplicated and tandem duplicated genes for each metabolic pathway (Table S4). Each pathway was tested for deviation from expected levels of gene duplication via χ^2^ analysis with the *P* values tested for significance under an FDR of 0.05 (Table S4).

### Sequence Diversity

A previously published dataset measuring genomic sequence diversity in 27 accessions was used to test for differential sequence diversity between metabolic pathways [52]. Estimates of Θ, π, and Tajima's D per gene were obtained from published data and the average across the genes within a metabolic pathway was calculated. Bootstrap analysis as described above was conducted to compare each metabolic pathways average Θ, π, and Tajima's D to a random genomic sample with a similar sized pathway. No pathways were observed to have a statistically significant bias in any sequence diversity value.

## Supporting Information

Table S1 Per Gene Statistical Values. The per gene statistical values are presented for all genes measured on the Affymetrix ATH1 microarray for Arabidopsis for the four datasets utilized in this manuscript. The datasets derived from experiments querying gene expression in response to abiotic stress variation, developmental variation, and two independent analysis of variation between different natural Arabidopsis accessions. Probe Set represents the probe set on the ATH1 microarray, AGI is the Arabidopsis gene code, Duplication represents the predicted gene duplication status [5]. Mean = mean transcript accumulation (μ) in log_2_ across the samples within the dataset, Var = per transcript variance (σ^2^) across the samples within the dataset, VMR = σ^2^/μ for each transcript, and CV = σ/μ for each transcript. For the dataset Accession I, R^2^ is the pearson correlation coefficient for transcript accumulation between the different accessions using all of the genes within a pathway.
(8.64 MB XLS)

Table S2 Biosynthetic Pathways. List of biosynthetic pathways and genes per each pathway utilized within this manuscript.
(0.20 MB XLS)

Table S3 Estimates of transcript variance per biosynthetic pathway. The mean statistical values per pathway and significance of the deviation from the genomic mean are presented. Pathway lists the biosynthetic pathway, N is the number of genes per pathway, Mean is the mean transcript accumulation in log_2_ across the genes within the pathway for the given dataset. VMR is the mean VMR across the genes within the pathway for the given dataset and P_VMR_ is the likelihood that this is equivalent to a random collection of N genes from the whole genome as estimated by 2000 random permutations. CV is the mean CV across the genes within the pathway for the given dataset and P_CV_ is the likelihood that this mean CV is equivalent to a random collection of N genes from the whole genome as estimated by 2000 random permutations.
(0.06 MB XLS)

Table S4 Duplication bias within metabolic pathways. The distribution of gene duplications within the pathways is presented. Genes (N) lists the number of genes within a pathway while Observed gives the number of these genes that are classified as segmental duplicates or tandem duplicates. The genomic frequency of segmental duplicates and tandem duplicates were used to predict the expected frequency of duplications for a given N. The fit of goodness between the observed and expected values were tested via χ^2^ and only P values significant at an FDR of 0.05 are presented as significant. This was done for both segmental duplicate and tandem duplicates.
(0.04 MB XLS)

Table S5 Sequence diversity within metabolic pathways. The distribution of sequence diversity estimates for the metabolic pathways are presented, θ, π, and Tajima's D. The per gene values for these paramaters were obtained from previously published data [52]. P values for deviation from the genomic average were obtained via permutation as described.
(0.05 MB XLS)
